# Lightweight Sewer Pipe Crack Detection Method Based on Amphibious Robot and Improved YOLOv8n

**DOI:** 10.3390/s24186112

**Published:** 2024-09-21

**Authors:** Zhenming Lv, Shaojiang Dong, Jingyao He, Bo Hu, Qingyi Liu, Honghang Wang

**Affiliations:** 1 School of Mechatronics and Vehicle Engineering, Chongqing Jiaotong University, Chongqing 400074, China; 2 Engineering Research Centre of Diagnosis Technology of Hydro-Construction, Chongqing Jiaotong University, Chongqing 400074, China; 3 Chongqing Institute of Surveying and Mapping Science and Technology, Chongqing 401120, China; 4 School of Electricity, Shanghai Dianji University, Shanghai 201306, China

**Keywords:** sewage pipe robot, lightweight, YOLOv8n, RGCSPELAN, Detect_LADH, LSKA, safe maintenance

## Abstract

Aiming at the problem of difficult crack detection in underground urban sewage pipelines, a lightweight sewage pipeline crack detection method based on sewage pipeline robots and improved YOLOv8n is proposed. The method uses pipeline robots as the equipment carrier to move rapidly and collect high-definition data of apparent diseases in sewage pipelines with both water and sludge media. The lightweight RGCSPELAN module is introduced to reduce the number of parameters while ensuring the detection performance. First, we replaced the lightweight detection head Detect_LADH to reduce the number of parameters and improve the feature extraction of modeled cracks. Finally, we added the LSKA module to the SPPF module to improve the robustness of YOLOv8n. Compared with YOLOv5n, YOLOv6n, YOLOv8n, RT-DETRr18, YOLOv9t, and YOLOv10n, the improved YOLOv8n has a smaller number of parameters of only 1.6 M. The FPS index reaches 261, which is good for real-time detection, and at the same time, the model also has a good detection accuracy. The validation of sewage pipe crack detection through real scenarios proves the feasibility of the proposed method, which has good results in targeting both small and long cracks. It shows potential in improving the safety maintenance, detection efficiency, and cost-effectiveness of urban sewage pipes.

## 1. Introduction

Underground municipal sewers are an indispensable component of urban infrastructure. They play a critical role in safeguarding public health [1,2,3] by preventing the spread of diseases [4,5] and protecting the natural environment from pollution [6,7,8]. These complex pipeline systems efficiently transport domestic and industrial wastewater away from urban areas to wastewater treatment plants, where it undergoes purification, thereby reducing the contamination of rivers, lakes, and groundwater. Furthermore, effective maintenance of sewer pipelines [9] is vital for extending their service life, enhancing wastewater treatment efficiency, lowering maintenance costs, and enabling prompt emergency responses. Regular inspection and maintenance not only improve the quality of life for residents but also ensure compliance with environmental regulations and support sustainable urban development [10,11,12].

Defect detection techniques for underground sewage pipes have been extensively researched and implemented, utilizing a range of methodologies such as closed-circuit television (CCTV), laser scanning, sonar detection, infrared thermography, and ultrasonic detection [13,14,15]. Among these, CCTV technology has demonstrated significant potential and commercial maturity for visual data collection due to its intuitive application, high efficiency, cost-effectiveness, and ease of operation [16]. This approach typically involves the deployment of robotic crawlers equipped with remote controls and video monitors to capture the internal conditions of the pipelines. Technicians subsequently analyze the video recordings to identify and assess defects within the sewer pipes [17,18].

However, the accuracy and efficiency of manual inspection are often constrained by the operator’s experience and skills, the working environment, and the clarity of the images [19]. This labor-intensive manual identification method, which is highly subjective and error-prone, is no longer able to meet the growing demand for pipeline defect detection and the high standard of accurate hydraulic model calibration. Therefore, more automated equipment and intelligent inspection techniques have been developed to improve the accuracy and efficiency of inspection. Robotic inspection methods for sewer pipe structures are now studied based on drones and pipe robots. Pandy et al. [20] used an automatic control mechanism to drive an unmanned aerial vehicle (UAV) to identify defects in municipal sewer pipelines. Pieczynski et al. [21] used an embedded UAV to process pipe cracks using deep learning-accelerated images. Vitry et al. [22] proposed a fully automated framework that utilizes high image overlap and a multi-view approach for UAV imaging measurements to improve detection performance. Nishida et al. [23] investigated the use of multiple UAVs for sewer pipe inspection. The use of multiple UAVs to establish a multi-hop wireless network inside the sewer pipe improves the detection accuracy. However, in the actual inspection process, drones often cause the problem of difficult detection due to signal loss, and at the same time, in the case of half-water pipelines or even more water, the risk of drone inspection is higher. In contrast to drones, sewage pipe robots have good stability and the ability to adapt to multiple working conditions.

Depending on the differences in the media within the sewer pipes in different geographical areas, they can be categorized as watered, silted, or dry pipes. Zhang et al. [24] used a wheeled sewer pipe robot with a cylinder inspection method to make the inspection process more robust. However, it is difficult to operate in watered pipes. Edwards et al. [25] used vision-based manhole recognition technology to achieve sewer pipe localization and crack detection in watered pipes. However, it can only move in watered pipes and cannot move in dry pipes. Lou et al. [26] developed a scalable differential screw-driven pipeline dredging robot, which proved through simulation that the robot is structurally sound, has the functions of cutting, mixing, and transporting silt, and can realize the mud flow operation state. However, it was not applied to a real inspection environment. Tirado et al. [27] used a limbless soft robot based on earthworm bionic technology for inspection in sewer pipes; however, it was only applicable to dry pipes. In summary, most of the current sewer pipe robots are only applicable to a single working condition, and sewer pipe robots for multiple working conditions have not yet been found.

Deep learning technology has become an important direction for current research and its application. The robot collects images of sewage pipe diseases, which can be processed by a deep learning model to improve detection accuracy and detection efficiency. Situ et al. [28] proposed a real-time detection method that combines a YOLOv5-based target detection network, transfer learning, and channel pruning techniques to address the specific characteristics and detection requirements of sewer pipe defects. The results demonstrated that the model offers significant advantages in practical engineering applications. Similarly, Wang et al. [29] introduced the PIPE-ConvNet model, which leverages a convolutional neural network for automatic surface anomaly detection in wastewater pipelines. Comparative results showed that this model excels in detecting pipe defects. C et al. [30] developed an automated framework for sewer pipe defect detection, integrating the attention mechanism, an improved YOLOv5 architecture, and positional information recognition from CCTV videos. Their real-time defect detection model achieved a mean average precision (mAP) of 75.9% on the proposed dataset. Nonetheless, the accuracy of these models requires further enhancement. He et al. [31] presented an automatic localization method that accurately determines the longitudinal distance of structural defects in sewers through pipe diameter image processing, based on a pinhole-based defect monocular ranging model. This approach improves the spatial localization accuracy of defects, providing a robust and stable solution. Xiao et al. [32] developed a comparative learning module to enhance the deep learning process for defect detection, significantly improving the average precision (AP) value of the defect detection model. Zuo et al. [33] employed a multi-scale feature fusion mechanism to integrate convolutional features at different scales, enriching both semantic and detail information to achieve accurate crack detection. Despite these advancements, the real-time detection performance of these methods remains inadequate. Wang et al. [34] proposed an intelligent damage detection method using a fine-tuned fully convolutional network (FCN) algorithm to segment and measure five types of defects in sewer pipes. Guo et al. [35] constructed an image sampling framework using a pipeline-extended feature pyramid network (P-EFPN) to capture more textures in the edge regions of images, thereby improving defect detection accuracy. However, the real-time performance of these methods is also lacking. Minh Dang et al. [36] introduced an efficient and robust framework for sewer defect localization to mitigate the cumbersome and time-consuming nature of human visual evaluation. Their model demonstrated superior performance experimentally, although it has a large number of parameters.

In summary, at present, domestic and foreign scholars have carried out research on sewage pipeline crack detection based on robotics and deep learning, and from the point of view of safety, high efficiency, real time, and the accuracy of the detection model, the above methods have not accomplished these tasks well, and there are still drawbacks in some aspects.

The remainder of this paper is organized as follows. The image acquisition system for the amphibious sewer pipe robot is described in Section 2. The design of the proposed deep learning model is described in Section 3. The dataset and the configuration of the experiment is described in Section 4. The experimental design and results are presented in Section 5. Conclusions are discussed in Section 6.

## 2. Vision Acquisition System Based on Sewage Pipe Robots

**Figure 1:**
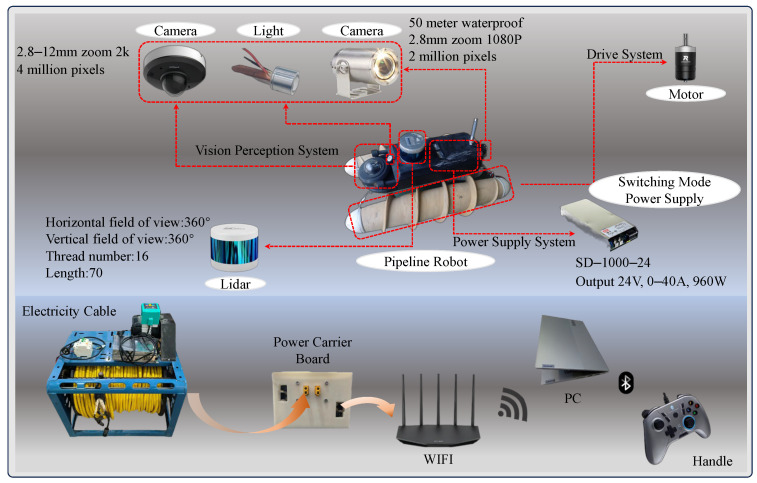
Sewage pipe robot overall structure.

In order to fulfill the need of apparent crack detection for many types of pipelines, a sewage pipeline robotic device equipped with an image acquisition system was designed to move through watered, dry, and silted pipelines to collect data for crack detection. Figure 1 shows the design of the key components of the proposed amphibious sewer pipe robot, which mainly consists of an image acquisition module, an amphibious movement module, and a motion control module.

### 2.1. Image Acquisition Module

The proposed image acquisition module includes a front-facing Dahua camera with a gimbal, a rear-facing waterproof camera with lights, and four self-developed large lumen lights.

The sewage pipe robot can be suspended on the water surface through its own buoyancy in the water pipeline and capture the crack information through its high-definition camera, which has stable and rapid imaging with good durability and stability. The selected Dahua 360° PTZ camera can capture the surroundings and observe cracks in any position of the pipeline. At the same time, the camera has a 2.8–12 mm zoom function, allowing it to clearly observe the fine details of the cracks. As its waterproof effect is poor, the use of a waterproof shell over the outside protects the camera from water. When the pipeline wall is darker, the visual perception effect is poor. The arrangement of 3 large lumen lights in the front left, right, and upper diagonal of the front camera ensures that all of the viewpoints have the same brightness; in addition, the front camera has a resolution of 4k, ensuring an optimal visual perception effect. The rear camera provides a rear view, which can be useful in the event of the pipeline robot being trapped in a narrow pipeline: the robot can be directly guided back to the pipeline mouth without risk of being stuck in the pipeline due to a too-small turn radius.

### 2.2. Amphibious Mobility Module

**Figure 2:**
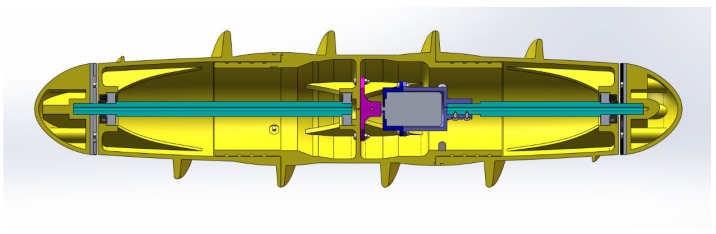
Internal structure of drive roller.

The robot mobile module is of Archimedean solenoidal design, gaining power through its rotation. The material was selected to be nylon and was processed using 3D printing technology. The overall structure includes fixed end caps for the shafts at both ends, crash disks, fixed connecting brackets, shafts, bearings, oil seal retaining rings, motors, motor flanges, and a three-section drum shell. In order to facilitate the assembly and processing, the drum spoke part was divided into three sections. Through the splicing of the two drums connected together, and splicing part of the O-ring seal to form a static seal, the drum and the shaft were connected through the bearings and oil sealing retaining ring to form a dynamic seal. The motor was fixed to the shaft and connected to the fixed end cover, while the output shaft of the motor was connected to the drum through the flange, so that the power could be transmitted from the motor to the drum. Both sides of the end caps should be bolted to the shaft, while the shaft and end caps connected to the motor should be reserved for threading holes. The end caps were bolted to the fixing bracket of the drum, and the drum was fixed to the robot body by the fixing bracket. The structure is shown in Figure 2.

### 2.3. Motion Control Module

**Figure 3:**
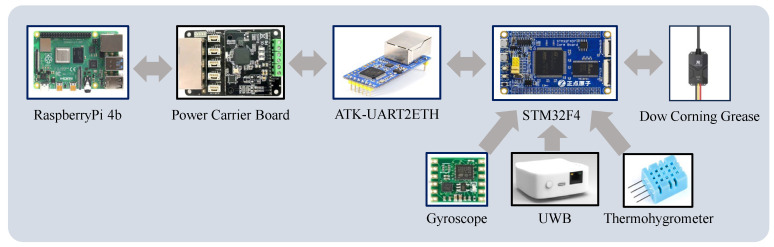
Electronic bin circuit system.

In order to realize movement and position changes inside the pipeline, the robot realizes multiple motion modes through differential control, including forward and backward, left and right steering, left and right traversing, and so on. The maximum speed of the robot is 1.5 m/s in silt and 1.0 m/s in water, and it takes only 3.2 s to complete its own in situ rotation; its traversing speed reaches 2 m/s in the dry pipe, and 1.2 m/s in some cases. The robot is driven by brushless motors to drive the drums. The power supply inside the pipeline robot is a 220 v–24 v switching power supply, as shown in Figure 3, and STM32F4 is used as the main control chip of the robot to drive the motors as well as a variety of sensors. At the same time, the pipeline robot realizes PID control through a gyroscope to keep the robot balanced in the dynamic water flow pipe and prevent tilting.

## 3. Materials and Methods

In silty, dry pipes there may be rocks and pits causing ruggedness in the pipe, and the amphibious sewage pipe robot will shake as it travels, causing difficulty in capturing cracks with the camera. Furthermore, there may also be water splashing, causing the camera to capture incorrectly, which is one of the main difficulties. Secondly, the environment inside the sewage pipe is dark; although large lumen lights are used as the main auxiliary visual perception equipment, for large pipes, cracks far away from the camera will be difficult to capture, and there will be missed detection. Even if they are captured, they may be blurry. Fast detection of cracks can reduce the detection leakage, so a real-time fast detection model is proposed.

### 3.1. YOLOv8 Algorithm

**Figure 4:**
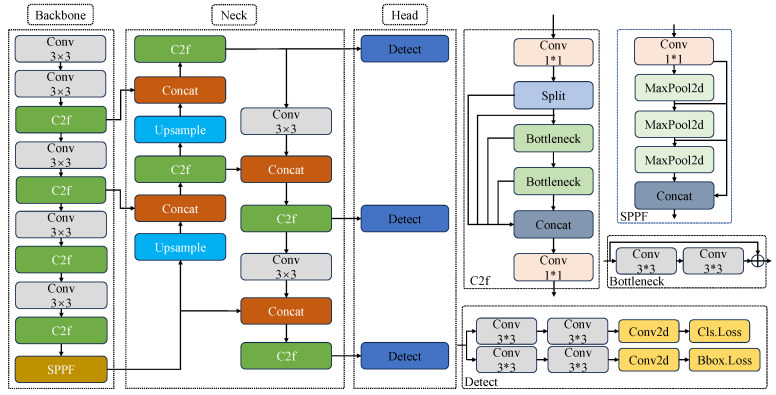
Original YOLOv8s framework.

YOLOv8 introduces a new and enhanced YOLO model, incorporating several innovations and improvements over the original design. These enhancements can be categorized into three main aspects: (1) Backbone network and neck improvement: Inspired by the design philosophy of YOLOv7 ELAN [37], the C3 structure from YOLOv5 is replaced with the C2f structure, which provides a richer gradient flow. Additionally, the number of channels is adjusted for different scale models, resulting in significant performance improvements. (2) Head structure: The model’s head is upgraded to a mainstream decoupled head structure and adopts an anchor-free approach. (3) Loss function enhancement: YOLOv8 abandons the previous IOU matching or unilateral proportion distribution method in favor of the task-aligned assigner for positive and negative sample matching. it also introduces the distribution focal loss (DFL). A variety of optimization modules are integrated to enhance the performance and efficiency of target detection. These modules include Upsample for enhancing feature details, C2f (confidence to focus) for improving confidence in target area detection, SPPF (spatial pyramid pooling with focus) for multi-scale feature fusion, MaxPool (maximum pooling) to reduce computational effort, and Bottleneck (bottle neck layer) to reduce the number of parameters. The integration of these techniques allows YOLOv8 to reduce computational complexity and resource consumption while maintaining high accuracy. The network structure is illustrated in Figure 4.

### 3.2. The Improved YOLOv8 Network

**Figure 5:**
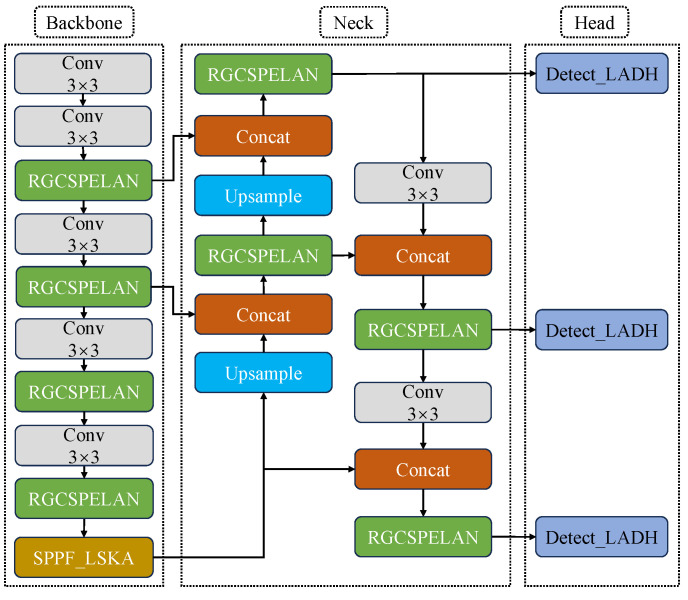
Framework of the improved YOLOv8n network.

**Figure 6:**
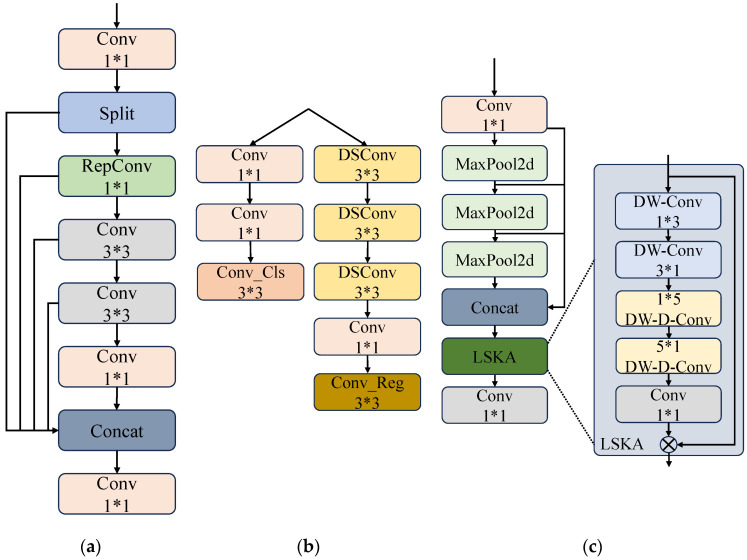
Improved modules in YOLOv8n: (**a**) RGCSPELAN, (**b**) Detect_LADH, and (**c**) SPPF_LSKA structure diagram.

To achieve high-precision and high-efficiency crack detection while reducing model size, a novel target detection algorithm RLL-YOLOv8 (with the integration of the RGCSPELAN module, SPPF-LSKA module, and LADH) based on the YOLOv8n network is proposed. The structure of this model is illustrated in Figure 5. To streamline the overall YOLOv8n model, the original C2f module was replaced with the RGCSPELAN module, as shown in Figure 6a. Inspired by the GhostNet [38] concept, which addresses the redundancy in intermediate feature mappings computed by mainstream CNNs, a simple operation was employed to generate a portion of the redundant feature maps. These feature maps were then combined to form a new output, effectively reducing computation and the number of parameters. Additionally, the Bottleneck module in the original C2f was discarded. To compensate for the performance loss caused by removing the residual fast, RepConv was utilized in the gradient circulation branch. This enhanced the model’s feature extraction and gradient circulation capabilities, with RepConv being fusible during inference.

The SPPF module is a core component of YOLOv8n, offering benefits in detection accuracy and speed. However, it also presents drawbacks, particularly regarding computational redundancy when handling features at different scales. To address these issues, the LSKA module [39] was introduced, as shown in Figure 6c. DW-Conv and DW-D-Conv are two kinds of convolutional operations. DW-Conv applies convolutional kernels independently on each input channel, which is suitable for extracting local features with high computational efficiency and fewer parameters. DW-D-Conv, on the other hand, increases the sensory field of the model through two deep convolutional operations, which is more suitable for capturing long-distance dependencies in images. By innovatively decomposing the two-dimensional convolution kernels of the deep convolutional layer into cascaded horizontal and vertical one-dimensional convolution kernels, it realizes the direct application of large convolution kernels in the attention module without adding extra computational burden. This design significantly reduces the computational complexity and memory footprint of the model, while enhancing the ability to capture long-range dependencies in images. In practice, the high performance of the LSKA module makes it possible to accurately recognize and capture fine pipe crack details, as well as shape features, thus demonstrating performance beyond existing models in vision tasks such as image classification, object detection, and semantic segmentation. This breakthrough provides visual attention networks with a more efficient and accurate tool when dealing with complex visual scenes. This is crucial for practical pipeline inspection scenarios, where LSKA’s robustness ensures stable crack detection under varying lighting conditions, background interference, and image noise.

Additionally, a lightweight detection head, LADH [40], was introduced in the YOLOv8n Head component. As shown in Figure 6b, Detect_LADH employs 3 × 3 depthwise separable convolution (DWConv) [41] to replace traditional convolution. This method reduces the number of parameters by decomposing the convolution operation into depthwise convolution and pointwise convolution. Unlike in traditional convolution where the number of input channels is multiplied by the number of output channels and then multiplied by the size of the convolution kernel, deep convolution handles each input channel independently, with the input channels multiplied by the size of the convolution kernel. While point-by-point convolution uses a 1 × 1 convolution kernel on the output of the depth convolution, with the number of parameters being the number of input channels multiplied by the number of output channels—an order of magnitude much smaller than the number of parameters in traditional convolution—point-by-point convolution merges the feature maps in the depth direction to effectively utilize the information between the channels. This approach not only enhances computational efficiency, making it suitable for resource-limited environments, but also improves detection accuracy by reducing parameter redundancy between tasks, resolving conflicts between classification and regression tasks, and enhancing feature extraction for crack details.

## 4. Datasets and Experiment Configurations Results

### 4.1. Dataset

The Sewer-ML dataset [42] was selected for training and initial evaluation of the proposed method. This publicly available dataset comprises annotated frames extracted from sewer inspection videos. Each annotation corresponds to a ground-truth label for a specific class at a designated second in the video and is associated with a specific location within the pipe. The dataset includes 75,618 annotated sewer inspection videos from three different Danish water companies, totaling 1.3 million images across 18 classes. For this study, the crack portion of the crack, rupture, and shedding categories was used, with each image sized at 352 × 288 pixels. The data encompass a wide range of materials, shapes, and sizes from both mains and laterals, reflecting the natural variations encountered in actual sewer inspections.

**Figure 7:**
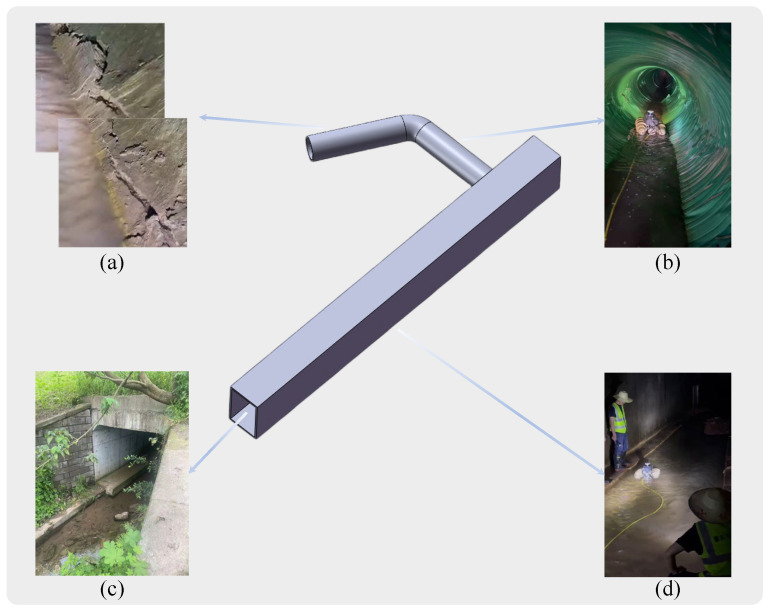
Sewer structure sketch: (**a**) sewer (concrete), (**b**) sewer (reinforced plastic), (**c**) entrance, (**d**) box culvert.

Approximately 4000 images of sewer pipes with cracks were selected and randomly divided into training, validation, and test sets in an 8:1:1 ratio. Specifically, 3200 images were used to train the sewer pipe crack detection network RLL-YOLOv8, with the remaining 400 images each designated for validation and testing. Field data collection took place inside a culvert near Fengge Road in Jiulongpo District, Chongqing City, as depicted in Figure 7. The culvert, featuring a box culvert structure, extends up to 30 m in length. It contains a cylindrical pipe, surrounded by silt and a small amount of water. The outer wall of the pipe’s first half comprises approximately 10 m of corrugated pipes, while the latter part consists of concrete. Due to prolonged exposure to vibrations from large trucks and constant water presence, the pipeline is prone to breakage and collapse. Pipeline robots were employed to inspect the internal condition of the pipeline, as shown in Figure 5. The captured images were inputted into the model for crack detection.

**Figure 8:**
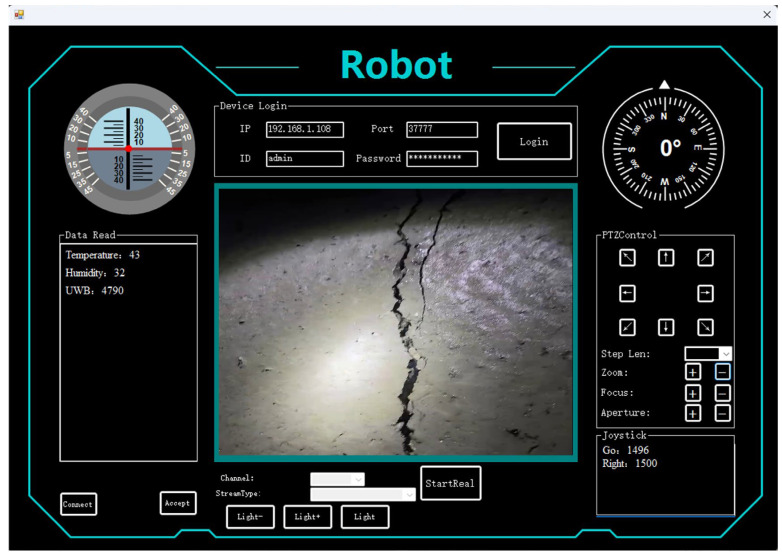
Sewage pipe robot uploader.

In the actual movement process, the camera uses an acquisition speed of 1 frame rate per second to collect data in the pipeline, and uses the C# language, Visual Studio software 2022, and Winform architecture to build a sewage pipeline robot uploader, as shown in Figure 8. Through the uploader, the operation of the robot can be viewed in real time. About 30 images of the collected cracks were input into the model to test the model effect of RLL-YOLOv8. The data used in testing the RLL-YOLOv8 model on the sewage pipe are shown in Table 1.

### 4.2. Dataset Annotation

**Figure 9:**
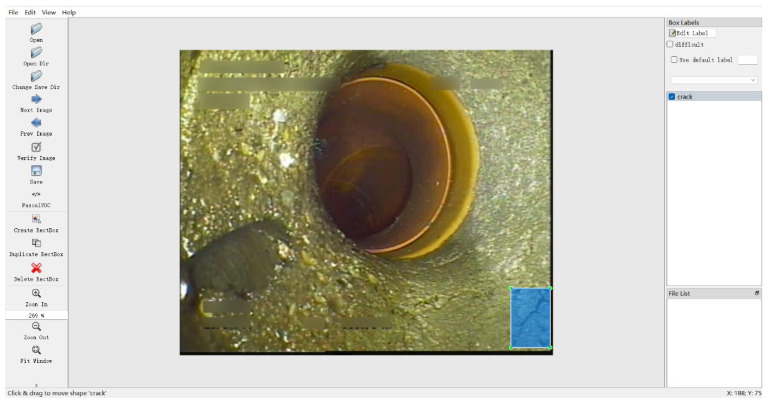
Example of data annotation.

The cracks in the data were labeled using the Labelimg script, as shown in Figure 9.

### 4.3. Experimental Configuration

This study was based on an Ultralytics framework, using the Ubuntu 20.04 operating system on a computer configured with 15vCPU Intel(R) Xeon(R) Platinum 8474P CPU@2.60GHz and NVIDIA RTX4090 (24 G). The modeling for this experiment was conducted a on PyTorch 1.10. 0, Cuda 11.3 framework. The size of the input image was 352 × 288, the number of training rounds was 300, the batch size was 32, the learning rate was 0.01, and the chosen optimization algorithm was stochastic gradient descent (SGD) [43] with momentum set to 0.937.

## 5. Experiments and Discussion

### 5.1. Evaluation Metrics

In terms of performance evaluation, Precision (*P*), Recall (*R*), mean average precision (mAP), number of parameters (Params, (M)), and frames per second (*FPS*) were used as key metrics. Precision (*P*) assesses the accuracy of the detection results by calculating the ratio of correctly recognized objects to the total number of detections, directly reflecting the algorithm’s accuracy. Recall (*R*) measures the algorithm’s ability to identify real existing objects, determined by the ratio of detected objects to the total number of actual objects. An increase in Recall indicates a reduction in the algorithm’s omission rate. True positive (*TP*) and true negative (*TN*) represent correctly classified positive and negative samples, respectively, while false positive (*FP*) and false negative (*FN*) denote misclassified negative and positive samples. Together, these metrics provide a comprehensive assessment of the algorithm’s performance. Precision and Recall are calculated as shown in Equation (1). Mean average precision (*mAP*) is a crucial measure of a target detection model’s performance, integrating Precision and Recall dimensions. In the sewer crack detection experiments, mAP50 and mAP50:95 are used as the final evaluation metrics; mAP50 represents the average precision at an IoU threshold of 0.5, and mAP50:95 is the average *mAP* obtained from 10 thresholds, ranging from 0.5 to 0.95 in steps of 0.05. The computational formula is shown in Equation (3). The *F*1 score, which is the harmonic mean of Precision and Recall, is calculated using the formula in Equation (4). *FPS* measures the inference time, calculated as shown in Equation (5), where preprocess denotes the image preprocessing time, inference is the inference time of the image, and postprocess is the post-processing time of the image, all measured in milliseconds.

(1) $$Precision=\frac{TP}{TP+FP}$$

(2) $$Recall=\frac{TP}{TP+FN}$$

(3) $$mAP=\frac{1}{k}\int_{0}^{1}P(R)dR$$

(4) $$F1=\frac{2\times P\times R}{P+R}$$

(5) $$FPS=\frac{1000}{preprocess+inference+postprocess}$$

### 5.2. Model Selection

The YOLOv8 series includes the YOLOv8n, YOLOv8s, YOLOv8m, YOLOv8l, and YOLOv8x, which maintain the same overall structure. The main differences are in the depth of the network structure, the number of layers, the size of the convolutional kernels, the downsampling vs. upsampling strategy, the complexity of the attention mechanism, the width of the network, and the use of residual connections. Lightweight versions (e.g., “n” and “s”) usually have fewer layers and smaller convolutional kernels to reduce computation and model size, while larger versions (e.g., “m”, “l”, and “x”) have more layers and larger convolutional kernels to capture richer feature information. These five models were trained for evaluation, and their performance was comprehensively assessed using metrics such as mAP50, F1 score, FPS, Params (M), and FLOPs (G). The real-time effect of model detection can be analyzed through FPS, where Params (M) indicates the number of parameters of the model and FLOPs (G) represents the number of computational resources consumed by the model. The results, shown in Table 2, indicate that YOLOv8n is the smallest and fastest model in the series. In contrast, YOLOv8s, YOLOv8m, YOLOv8l, and YOLOv8x, while potentially superior in detection performance, have larger model sizes and higher computational resource requirements, which may pose deployment challenges. Considering all factors, YOLOv8n was selected as the base model for this study.

### 5.3. Model Benchmarking Experiments

To validate the suitability of the YOLOv8n model as a benchmark for sewer crack detection, a series of benchmark experiments were conducted. These experiments compared the detection accuracy, model lightweight, and detection efficiency of various algorithms, including YOLOv5n, YOLOv6n [44], YOLOv8n, RT-DETRr18 [45], YOLOv9t [46], and YOLOv10n [47]. All models were evaluated using the same training and validation sets, as shown in Table 3.

In terms of detection accuracy, the YOLOv8n model demonstrated exceptional performance. While the proposed model’s Precision index ranked second, just 0.006 lower than the top-performing YOLOv10n algorithm, it achieved the highest Recall index, surpassing the second-best model by 0.011. Notably, YOLOv8n secured the top position in both the F1 score and mAP, underscoring its superior detection accuracy and justifying its selection as a benchmark model.

Regarding detection efficiency and model parameter count, YOLOv5n and YOLOv9t consumed fewer computational resources, with YOLOv8n following closely behind. YOLOv8n’s parameter count was 3.1 M, just 1.1 M higher than YOLOv9t, which had the lowest parameter count. However, YOLOv8n excelled in FPS, outperforming the second-place model by 48 FPS. Although YOLOv9t slightly outperformed YOLOv8n in terms of parameter count and computational resource consumption, its FPS was significantly lower.

Conversely, while YOLOv10n had a comparable parameter count and computational resource consumption to YOLOv8n, the latter’s superior FPS metric compensated for its relatively weaker metrics.

### 5.4. Comparison with Other Methods on the Sewer-ML Dataset

**Figure 10:**
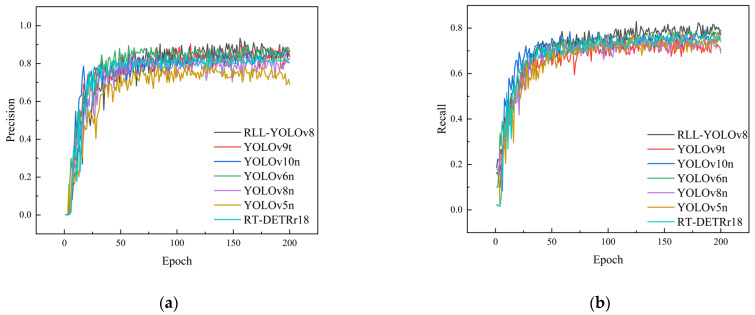

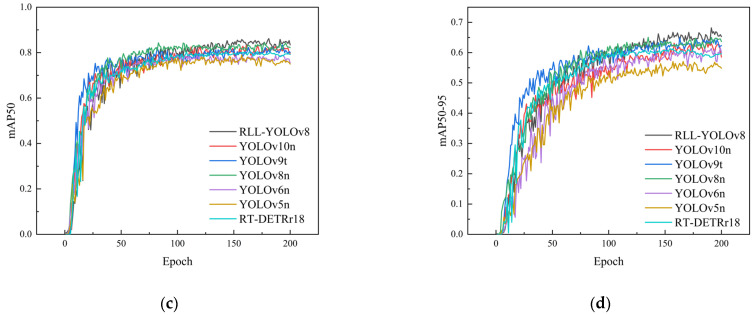
Metric comparisons of several algorithms on the Sewer-ML dataset: (**a**) curve of Precision, (**b**) curve of Recall, (**c**) curve of mAP50, and (**d**) curve of mAP50-95.

**Figure 11:**
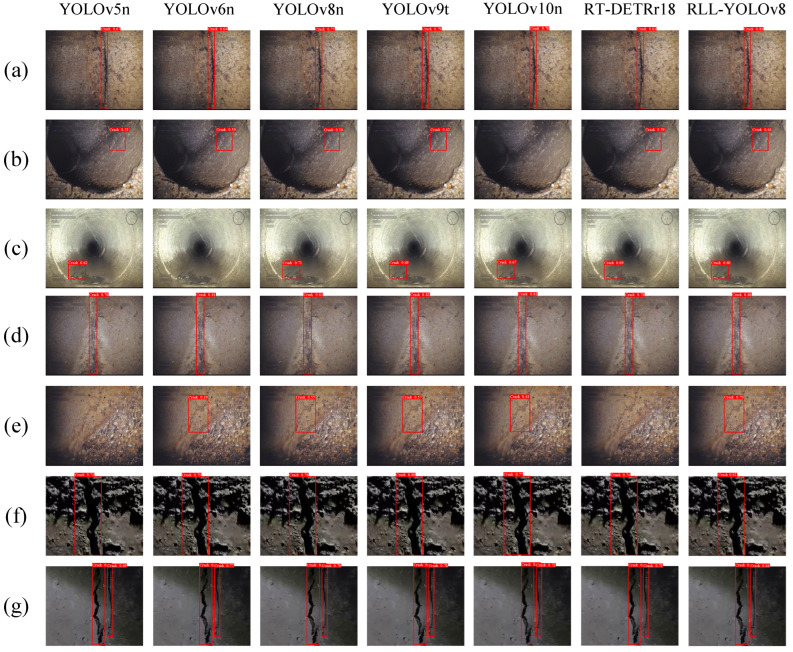
The results of the cracking dataset on different models. (**a**–**e**) are the samples from the dataset. (**f**,**g**) are the samples of data from real collection.

In this study, the RLL-YOLOv8 sewer pipe crack detection model was compared with several other object detection methods, including YOLOv5n, YOLOv6n, YOLOv8, RT-DETRr18, YOLOv9t, and YOLOv10n, to validate its effectiveness. YOLOv5n adopts anchor-based design and uses an efficient network structure, including CSPDarknet as the backbone network and PANet (path aggregation network) as the detection head. YOLOv6n introduces a bi-directional crosstalk module and anchor-assisted training to optimize the backbone and neck design to enhance the detection performance. YOLOv9t introduces PGI and GELAN to optimize the information flow and parameter efficiency, which improves the computational efficiency and parameter efficiency of the model, and reaches a new level of real-time target detection. YOLOv10n introduces a consistent dual allocation strategy to eliminate the dependence on NMS, and also enhances the feature extraction capability by using the large kernel convolution and partial self-attention mechanism, which improves the model performance. RT-DETR adopts the transformer architecture, which omits the anchor frame and NMS, and directly predicts the target to optimize the global information capture and detection efficiency. The same hyperparameter settings were applied across all models. The training results are depicted in Figure 10a–d, the experimental results are summarized in Table 3, and the comparison of each model is illustrated in Figure 11. Figure 10a–d shows the accuracy, Recall, mAP50, and mAP50-95 curves of several models during the training process. As observed in Figure 10a,b, the accuracy and Recall of RLL-YOLOv8 are superior throughout the training period. The mAP50 and mAP50-95 metrics are also higher for RLL-YOLOv8 compared to the other methods, indicating its enhanced performance and superior evaluation indices. These results suggest that RLL-YOLOv8 outperforms other methods and is well-suited for use as a sewer pipe crack detection model, offering higher accuracy and Recall, as well as better overall evaluation metrics. Considering model detection accuracy, lightweight design, and detection efficiency, YOLOv8n emerges as a reasonable and effective choice as a benchmark model for sewer crack detection.

From Table 3, it is evident that the accuracy of YOLOv5n and YOLOv6n is 81.0% and 83.8%, respectively, while the YOLOv8n, YOLOv9t, and YOLOv10n algorithms achieve around 90% accuracy. In comparison, RLL-YOLOv8 demonstrates a higher accuracy of 93.3%, outperforming the second-best model by 3%. In the mAP50 and mAP50-95 metrics, RLL-YOLOv8 also shows a significant advantage, exceeding the second-best results by 1.9% and 3.1%, respectively. This further confirms that RLL-YOLOv8 is more precise in detecting sewer cracks. Regarding real-time performance and the number of parameters, RLL-YOLOv8 achieves the highest score, with an FPS of 261, which is 38 higher than the second-best model. Additionally, the number of parameters is only 1.6 M, which is 0.4 M lower than the second-best model. Overall, the proposed method in this paper is the one with the smallest number of parameters, meeting the requirements for real-time detection of sewer cracks while maintaining the highest accuracy.

Two long cracks, three fine cracks, and two real cracks were randomly selected for testing with comparison models such as RLL-YOLOv8. As shown in Figure 11, various models can accurately recognize long cracks in the downspout. From the two long cracks in Figure 11a,d and the real downspout cracks in Figure 11f,g, RLL-YOLOv8 achieves the highest recognition accuracy of 83%, 88%, 82%, and 84%. For fine cracks, some algorithms fail to recognize them effectively, with YOLOv5n, YOLOv6n, and YOLOv10n missing detections. Although YOLOv8n, YOLOv9t, and RT-DETRr18 can successfully detect fine cracks, their accuracy is not as high as that of RLL-YOLOv8. Compared with several other models, RLL-YOLOv8 has the highest accuracy. YOLOv8 shows the best performance overall, with good results on both long and small cracks.

These results highlight the superior performance of RLL-YOLOv8 in terms of accuracy, real-time detection, and parameter efficiency, making it the most suitable model for sewer crack detection.

### 5.5. Visualization and Interpretation Techniques for Sewer Pipe Crack Detection Models

**Figure 12:**
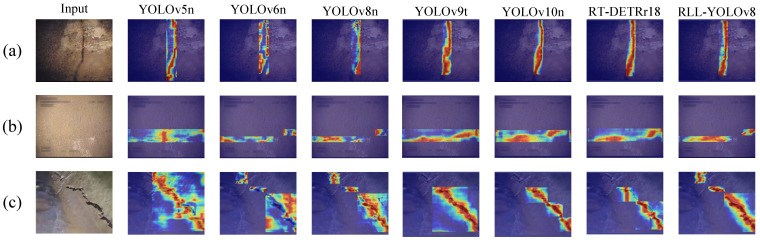
Comparison of several algorithmic heat maps. (**a**,**b**) are the samples from the dataset. (**c**) are the samples of data from real collection.

Multi-algorithm comparison experiments have confirmed that RLL-YOLOv8 excels in detecting sewer pipe cracks. To gain a deeper understanding of the intrinsic mechanisms of RLL-YOLOv8 and other comparison algorithms in the feature extraction process, a series of experiments were designed to visualize the attention heatmap. Grad-CAM++ (gradient-weighted class activation mapping) [48] was employed to create heatmap matrices to visualize and understand how deep learning models focus on important features in the input image when performing predictions. With Grad-CAM++, it is possible to visualize whether the model captures the key features of the cracks and highlights the pixel regions that have the greatest impact on the model’s output in the form of heatmaps. This visualization strategy not only enhances the understanding of the model’s decision-making process but also provides strong visual support for further model optimization. In the task of sewer pipe crack detection, the attention heatmap serves as an intuitive tool to effectively demonstrate the model’s focus on different regions in the image. Through this heatmap, it becomes clear how the model allocates its attention. Specifically, the shade of the color represents the importance of each location in the image: the darker the color, the stronger the positive response of the model to the region, indicating higher attention. As shown in Figure 12, background regions are usually represented in blue, indicating minimal model attention. Conversely, crack regions are presented in orange or red, highlighting the model’s significant attention to these areas. This intuitive representation quickly identifies the regions the model focuses on when processing the image. From Figure 12a, it can be observed that YOLOv5n pays more attention to the lower part of long cracks, with the upper part of the cracks more on the wall. YOLOv6n detects sewer pipe cracks but focuses on the wall at the edge of the cracks. YOLOv8n distributes its attention evenly across the cracks. YOLOv9t, YOLOv10n, and RT-DETRr18 can focus well on the downspout cracks, but RLL-YOLOv8 is more accurate in terms of edge contour, with the orange-red area better aligning with the overall crack contour. Figure 12b depicts a fine discontinuous crack. For this type of crack, YOLOv5n, YOLOv9t, YOLOv10n, and RT-DETRr18 detect two segments of discontinuous cracks within a single detection frame, resulting in extraneous sewer pipe wall information being attended to, causing inefficiency. YOLOv6n and YOLOv8n recognize two segments of cracks, but YOLOv6n still focuses more on the wall at the edge of the cracks, and YOLOv8n incompletely attends to one segment of the cracks. In contrast, RLL-YOLOv8 effectively focuses on both long and short segments of the cracks. Figure 12c shows sewer pipe crack data collected by the sewer pipe robot. YOLOv9t, YOLOv10n, and RT-DETRr18 exhibit missed detections, while YOLOv5n, YOLOv6n, and YOLOv8n can attend to the crack information. However, the red areas indicate attention to the sewer pipe wall surface, from which the conclusion can be drawn that RLL-YOLOv8 performs well in distinguishing the target from the background. It accurately identifies the crack region in the image due to its comprehensive feature extraction capability, maintaining high detection accuracy in complex and realistic backgrounds. The improved RLL-YOLOv8 model has significantly enhanced its focus on cracks and effectively distinguishes cracks from the background wall, making it more suitable for real-world application scenarios.

### 5.6. Ablative Studies

To evaluate the contribution of each module in the RLL-YOLOv8 model, a series of experiments were conducted to verify the impact of each module on detection performance. The results, presented in Table 4 and Table 5, reveal significant insights. When replacing the C2f module in YOLOv8n with the RGCSPELAN module, the Precision improved to 92.4%, Recall to 80.9%, mAP50 to 85.7%, and mAP50-95 to 67.3%, representing increases of 2.7%, 1.4%, 1.4%, and 2.2%, respectively, compared to the benchmark model. This improvement is attributed to the superior feature extraction and gradient circulation capabilities of the RGCSPELAN module. Introducing the Detect_LADH module resulted in a Precision of 90.6%, Recall of 80.1%, mAP50 of 85.1%, and mAP50-95 of 66.9%, improving the benchmark model by 0.9%, 0.6%, 0.8%, and 1.8%, respectively. The depth-separable convolution employed by the Detect_LADH module enhances the extraction of sewer crack features, thereby improving detection performance. Replacing the original SPPF module with the SPPF_LSKA module yielded a Precision of 91.2%, Recall of 82.1%, mAP50 of 85.4%, and mAP50-95 of 67.2%, showing improvements of 1.5%, 2.6%, 1.1%, and 2.1% over YOLOv8n. This enhancement is due to the LSKA module’s superior ability to capture crack details. Combining the three modules effectively resulted in improved detection accuracy and more precise identification of sewer pipe cracks.

In terms of model lightweighting and real-time detection performance, adding the RGCSPELAN module resulted in 2.3 M parameters, 167 FPS, and 6.9 Flops (G). Adding the Detect_LADH module yielded 2.4 M parameters, 156 FPS, and 7.4 Flops (G). After integrating the SPPF_LSKA module, the parameters increased to 3.4 M, FPS decreased to 111, and Flops (G) rose to 8.8. Despite the increase in parameters and decrease in FPS with the SPPF_LSKA module, the model still met real-time detection requirements. The effective combination of the three modules reduced the number of parameters to 1.6 M, increased FPS to 261, and decreased Flops (G) to 4.0.

In summary, the effective fusion of the three modules in the YOLOv8n model significantly enhances the accuracy and real-time performance of sewer pipe crack detection while maintaining a lightweight model.

## 6. Conclusions

This paper proposes a lightweight sewage pipe crack detection method based on a sewage pipe robot and improved YOLOv8n, which was proven to be feasible through a large number of experiments and successfully applied to an actual sewage pipe inspection project, with the main conclusions as follows:

(1) The designed amphibious sewage pipe robot, equipped with an image acquisition system, could efficiently move and detect cracks for sewage pipes in various working conditions.
(2) A lightweight sewer pipe crack detection model was proposed, based on an improved YOLOv8n to detect sewer pipe cracks in real time.
(3) The RGCSPELAN lightweight module was used to replace C2f, as well as the lightweight detection head Detect_LADH, which reduced the number of model parameters and at the same time ensured the model’s ability to extract features of sewer pipe cracks. In addition, the lightweight LSKA attention mechanism was added to the SPPF module to accurately capture the details and shape features of the pipe cracks without excessively increasing the number of model parameters.
(4) A large number of experiments were conducted on the publicly available dataset Sewer-ML and real sewer pipe data collected by sewer pipe robots to verify the impact of several improved modules on the effectiveness of sewer pipe crack detection. Compared with several more advanced detection algorithms, the proposed method had the best detection effect.

The use of amphibious sewage pipe robots can be fully adapted to the environment of sewage pipes with multiple working conditions, and promote the development of existing sewage pipe detection technology and equipment. The next steps will be to address the problem of unclear positioning of cracks, explore the use of high-precision LiDAR, the real-time construction of sewage pipeline cloud maps, and a method to detect the corresponding crack locations. At the same time, the use of multibeam sonar for crack detection could be considered, as well as a combination of multiple sensors under multiple working conditions; this could then complete the realization of overall pipeline crack detection.

## Figures and Tables

**Table 1:** RLL-YOLOv8 algorithmic dataset allocation.

Method	Training	Validation	Test	Total
RLL-YOLOv8	3200	400	400 + 30	4030

**Table 2:** Comparison of different models of YOLOv8. (Bold indicates highest score).

Model	mAP50	F1	FPS	Params (M)	FLOPs (G)
YOLOv8n	82.1	**84.3**	**223**	**3.1**	8.9
YOLOv8s	**83.3**	82.7	197	11.1	28.6
YOLOv8m	83.1	84.1	102	25.9	79.3
YOLOv8l	82.8	82.8	80	43.7	165.2
YOLOv8x	83.9	83.3	73	68.2	**257.8**

**Table 3:** Comparison results with other algorithms. (Bold indicates highest score).

Method	P	R	F1	mAP50	mAP50-95	Params	FPS	Flops (G)
YOLOv5n	0.810	0.782	0.796	0.780	0.571	2.7	143	7.8
YOLOv6n	0.838	0.783	0.810	0.792	0.616	4.5	102	13.1
YOLOv8n	0.897	0.795	0.843	0.843	0.651	3.1	223	8.9
YOLOv9t	0.883	0.784	0.831	0.834	0.650	2.0	97	7.8
YOLOv10n	0.903	0.763	0.827	0.831	0.633	2.7	175	8.4
RT-DETRr18	0.852	0.764	0.806	0.804	0.615	2.9	86	105.5
RLL-YOLOv8	**0.933**	**0.829**	**0.878**	**0.862**	**0.682**	**1.6**	**261**	**4.0**

**Table 4:** Comparison of detection accuracy ablation experiments (best scores are in bold).

Baseline	RGCSPELAN	LADH	LSKA	P	R	mAP50	mAP50-95
√				0.897	0.795	0.843	0.651
√	√			0.924	0.809	0.857	0.673
√		√		0.906	0.801	0.851	0.669
√			√	0.912	0.821	0.854	0.672
√	√	√		0.921	0.798	0.855	0.663
√	√		√	0.899	0.803	0.852	0.659
√		√	√	0.901	0.816	0.849	0.655
√	√	√	√	**0.933**	**0.829**	**0.862**	**0.682**

**Table 5:** Comparison of real-time and lightweight ablation experiments (best scores are in bold).

Baseline	RGCSPELAN	LADH	LSKA	Params(M)	FPS	Flops (G)
√				2.7	143	7.8
√	√			2.3	167	6.9
√		√		2.4	156	7.4
√			√	3.4	111	8.8
√	√	√		1.7	250	4.1
√	√		√	2.6	158	7.2
√		√	√	2.7	152	6.6
√	√	√	√	**1.6**	**261**	**4.0**

## Data Availability

The authors do not have permission to share data.

## References

[1] Blanco-Guzmán R., Atenciano-Crespillo J. (2024). Urban Sanitation in al-Andalus: The Case of Qurṭuba (Tenth to Thirteenth Century). Al-Masāq.

[2] Utepov Y., Neftissov A., Mkilima T., Shakhmov Z., Akhazhanov S., Kazkeyev A., Mukhamejanova A.T., Kozhas A.K. (2024). Advancing sanitary surveillance: Innovating a live-feed sewer monitoring framework for effective water level and chamber cover detections. Heliyon.

[3] Taweesan A., Koottatep T., Kanabkaew T., Polprasert C. (2024). Application of machine learning in sanitation management prediction: Approaches for achieving sustainable development goals. Environ. Sustain. Indic..

[4] Lin N., Zhang B., Shi R., Gao Y., Wang Z., Ling Z., Tian Y. (2024). Decay pattern of SARS-CoV-2 RNA surface contamination in real residences. Sci. Rep..

[5] Yao Y., Zhu Y., Nogueira R., Klawonn F., Wallner M. (2024). Optimal Selection of Sampling Points within Sewer Networks for Wastewater-Based Epidemiology Applications. Methods Protoc..

[6] Hong M., Niu D., Fu Q., Hui Z., Wan Z. (2024). Insights into bio-deterioration of concrete exposed to sewer environment: A case study. Constr. Build. Mater..

[7] Choi I., Lee H., Shin J., Kim H. (2012). Evaluation of the effectiveness of five odor reducing agents for sewer system odors using an on-line total reduced sulfur analyzer. Sensors.

[8] Zuo Z., Xing Y., Liu T., Zheng M., Lu X., Chen Y., Jiang G., Liang P., Huang X., Liu Y. (2024). Methane mitigation via the nitrite-DAMO process induced by nitrate dosing in sewers. Water Res..

[9] Hajare R., Labhasetwar P., Nagarnaik P. (2021). Assessment of Health Risk and Detailed Evaluation of Causative Factors Associated with Use of Contaminated Groundwater in the Remote Atolls. Water Air Soil Pollut..

[10] Li Y., Wang H., Dang L.M., Song H.-K., Moon H. (2022). Vision-based defect inspection and condition assessment for sewer pipes: A comprehensive survey. Sensors.

[11] Duque N., Scholten L., Maurer M. (2024). Exploring transitions of sewer wastewater infrastructure towards decentralisation using the modular model TURN-Sewers. Water Res..

[12] Jean M.-E., Morin C., Ossa J.E.O., Duchesne S., Pelletier G., Pleau M. (2024). Optimal distribution of green and grey infrastructures coupled with real time control of the sewer for combined sewer overflows control as an adaptation measure to climate change. Urban Water J..

[13] Hawari A., Alamin M., Alkadour F., Elmasry M., Zayed T. (2018). Automated defect detection tool for closed circuit television (cctv) inspected sewer pipelines. Autom. Constr..

[14] Liu Z., Kleiner Y. (2013). State of the art review of inspection technologies for condition assessment of water pipes. Measurement.

[15] Mukherjee S., Zhang R., Alzuhiri M., Rao V.V., Udpa L., Deng Y. (2022). Inline Pipeline Inspection Using Hybrid Deep Learning Aided Endoscopic Laser Profiling. J. Nondestruct. Eval..

[16] Wang M., Kumar S.S., Cheng J.C.P. (2021). Automated sewer pipe defect tracking in CCTV videos based on defect detection and metric learning. Autom. Constr..

[17] Kumar S.S., Wang M., Abraham D.M., Jahanshahi M.R., Iseley T., Cheng J.C.P. (2020). Deep Learning-Based Automated Detection of Sewer Defects in CCTV Videos. J. Comput. Civ. Eng..

[18] Meijer D., Scholten L., Clemens F., Knobbe A. (2019). A defect classification methodology for sewer image sets with convolutional neural networks. Autom. Constr..

[19] Haurum J.B., Moeslund T.B. (2020). A Survey on Image-Based Automation of CCTV and SSET Sewer Inspections. Autom. Constr..

[20] Pandey B.K., Pandey D., Sahani S.K. (2024). Autopilot control unmanned aerial vehicle system for sewage defect detection using deep learning. Eng. Rep..

[21] Pieczyński D., Ptak B., Kraft M., Piechocki M., Aszkowski P. (2024). A fast, lightweight deep learning vision pipeline for autonomous UAV landing support with added robustness. Eng. Appl. Artif. Intell..

[22] Vitry M.M., Schindler K., Rieckermann J., Leitão J.P. (2018). Sewer inlet localization in UAV image clouds: Improving performance with multiview detection. Remote Sens..

[23] Nishida A., Pham T.V., Ishihara S. (2024). Evaluation of An Intermittent Packet Transmission Method in UAVs-Based Sewer Pipe Inspection. Proceedings of the 2024 IEEE International Conference on Pervasive Computing and Communications Workshops and other Affiliated Events (PerCom Workshops).

[24] Zhang R., Worley R., Edwards S., Aitken J., Anderson S.R., Mihaylova L. (2023). Visual Simultaneous Localization and Mapping for Sewer Pipe Networks Leveraging Cylindrical Regularity. IEEE Robot. Autom. Lett..

[25] Edwards S., Zhang R., Worley R., Mihaylova L., Aitken J., Anderson S.R. (2023). A robust method for approximate visual robot localization in feature-sparse sewer pipes. Front. Robot. AI.

[26] Lou F., Guan J., Lu W. (2023). Design and analysis of extensible differential-speed helical drive pipe dredging robot. J. Eng..

[27] Tirado J., Jørgensen J., Rafsanjani A. (2023). Earthworm-inspired multimodal soft actuators. Proceedings of the 2023 IEEE International Conference on Soft Robotics (RoboSoft).

[28] Situ Z., Teng S., Liao X., Chen G., Zhou Q. (2024). Real-time sewer defect detection based on YOLO network, transfer learning, and channel pruning algorithm. J. Civ. Struct. Health Monit..

[29] Wang X., Thiyagarajan K., Kodagoda S., Zhang M. (2023). PIPE-CovNet: Automatic In-Pipe Wastewater Infrastructure Surface Abnormality Detection Using Convolutional Neural Network. IEEE Sens. Lett..

[30] Oh C., Dang L.M., Han D., Moon H. (2022). Robust Sewer Defect Detection With Text Analysis Based on Deep Learning. IEEE Access.

[31] He J., Hou Z., Zhu D., Li Z., Li Z. (2022). Automatic accurate longitudinal location of structural defects in sewer pipes via monocular ranging. Appl. Opt..

[32] Siu C., Wang M., Cheng J.C.P. (2022). A framework for synthetic image generation and augmentation for improving automatic sewer pipe defect detection. Autom. Constr..

[33] Zuo X., Ma B., Shen J., Shan Y., Khaleghian H. (2024). Mask-Guided Attention for Subcategory-Level Sewer Pipe Crack Classification. J. Pipeline Syst. Eng. Pract..

[34] Wang N., Fang H., Xue B., Wu R., Fang R., Hu Q., Lv Y. (2023). Automatic Damage Segmentation Framework for Buried Sewer Pipes Based on Machine Vision: Case Study of Sewer Pipes in Zhengzhou, China. J. Infrastruct. Syst..

[35] Guo W., Zhang X., Zhang D., Chen Z., Zhou B., Huang D., Li Q. (2022). Detection and classification of pipe defects based on pipe-extended feature pyramid network. Autom. Constr..

[36] Dang L.M., Wang H., Li Y., Nguyen T.N., Moon H. (2022). DefectTR: End-to-end defect detection for sewage networks using a transformer. Constr. Build. Mater..

[37] Wang C.-Y., Bochkovskiy A., Liao H.-Y.M. YOLOv7: Trainable bag-of-freebies sets new state-of-the-art for real-time object detectors. Proceedings of the 2023 IEEE/CVF Conference on Computer Vision and Pattern Recognition (CVPR).

[38] Han K., Wang Y., Tian Q., Guo J., Xu C., Xu C. GhostNet: More Features from Cheap Operations. Proceedings of the IEEE/CVF Conference on Computer Vision and Pattern Recognition (CVPR).

[39] Lau K.W., Po L.-M., Rehman Y.A.U. (2024). Large Separable Kernel Attention: Rethinking the Large Kernel Attention design in CNN. Expert Syst. Appl..

[40] Zhang J., Chen Z., Yan G., Wang Y., Hu B. (2023). Faster and Lightweight: An Improved YOLOv5 Object Detector for Remote Sensing Images. Remote Sens..

[41] Chollet F. Xception: Deep Learning with Depthwise Separable Convolutions. Proceedings of the 30th IEEE/CVF Conference on Computer Vision and Pattern Recognition (CVPR).

[42] Haurum J.B., Moeslund T.B. Sewer-ML: A Multi-Label Sewer Defect Classification Dataset and Benchmark. Proceedings of the IEEE/CVF Conference on Computer Vision and Pattern Recognition (CVPR).

[43] Mandt S., Hoffman M.D., Blei D.M. (2017). Stochastic Gradient Descent as Approximate Bayesian Inference. J. Mach. Learn. Res..

[44] Li C., Li L., Jiang H., Weng K., Geng Y., Li L., Wei X. (2022). YOLOv6: A single-stage object detection framework for industrial applications. arXiv.

[45] Zhao Y., Lv W., Xu S., Wei J., Wang G., Dang Q., Chen J. Detrs beat yolos on real-time object detection. Proceedings of the IEEE/CVF Conference on Computer Vision and Pattern Recognition.

[46] Wang C.Y., Yeh I.H., Liao HY M. (2024). Yolov9: Learning what you want to learn using programmable gradient information. arXiv.

[47] Wang A., Chen H., Liu L., Chen K., Lin Z., Han J., Ding G. (2024). Yolov10: Real-time end-to-end object detection. arXiv.

[48] Chattopadhay A., Sarkar A., Howlader P., Balasubramanian V.N. Grad-CAM plus plus: Generalized Gradient-based Visual Explanations for Deep Convolutional Networks. Proceedings of the 18th IEEE Winter Conference on Applications of Computer Vision (WACV).

